# Comparing computer-assisted learning activities for learning clinical neuroscience: a randomized control trial

**DOI:** 10.1186/s12909-022-03578-2

**Published:** 2022-07-03

**Authors:** Kiran Kasper Rajan, Anand S Pandit

**Affiliations:** 1 Bristol Medical School (PHS), University of Bristol, Canynge Hall, 39 Whatley Road, Bristol, BS8 2PS UK; 2 GKT School of Medical Education, King’s College London, London, UK; 3 Victor Horsley Department of Neurosurgery, National Hospital for Neurology and Neurosurgery, Queen Square, London, WC1N 3BG UK

**Keywords:** Computer-aided instruction, E-learning, Medical student, Neuroanatomy, Neuroscience, Teaching of neuroscience/neuroanatomy

## Abstract

**Background:**

Computer-assisted learning has been suggested to improve enjoyment and learning efficacy in medical education and more specifically, in neuroscience. These range from text-based websites to interactive electronic modules (eModules). It remains uncertain how these can best be implemented. To assess the effects of interactivity on learning perceptions and efficacy, we compared the utility of an eModule using virtual clinical cases and graphics against a Wikipedia-like page of matching content to teach clinical neuroscience: fundamentals of stroke and cerebrovascular anatomy.

**Methods:**

A randomized control trial of using an interactive eModule versus a Wikipedia-like page without interactivity was performed. Participants remotely accessed their allocated learning activity once, for approximately 30 min. The primary outcome was the difference in perceptions on enjoyability, engagement and usefulness. The secondary outcome was the difference in learning efficacy between the two learning activities. These were assessed using a Likert-scale survey and two knowledge quizzes: one immediately after the learning activity and one repeated eight weeks later. Assessments were analysed using Mann–Whitney U and T-tests respectively.

**Results:**

Thirty-two medical students participated: allocated evenly between the two groups through randomisation. The eModule was perceived as significantly more engaging (*p* = 0.0005), useful (*p* = 0.01) and enjoyable (*p* = 0.001) by students, with the main contributing factors being interactivity and clinical cases. After both learning activities, there was a significant decrease between the first and second quiz scores for both the eModule group (-16%, *p* = 0.001) and Wikipedia group (-17%, *p* = 0.003). There was no significant difference in quiz scores between the eModule and Wikipedia groups immediately afterwards (86% vs 85%, *p* = 0.8) or after eight weeks (71% vs 68%, *p* = 0.7).

**Conclusion:**

Our study shows that increased student satisfaction associated with interactive computer-assisted learning in the form of an eModule does not translate into increased learning efficacy as compared to using a Wikipedia-like webpage. This suggests the matched content of the passive webpage provides a similar learning efficacy. Still, eModules can help motivate self-directed learners and overcome the perceived difficulty associated with neuroscience. As computer assisted learning continues to rapidly expand among medical schools, we suggest educators critically evaluate the usage and cost–benefit of eModules.

**Supplementary Information:**

The online version contains supplementary material available at 10.1186/s12909-022-03578-2.

## Background

Digitalization of medical education has meant the development of different forms of computer-assisted learning (CAL)—defined as the use of any computer software to deliver or facilitate a learning experience [1]. One of the main proposed benefits of CAL is its flexibility and convenience [2]. It forms an important role in medical education [2, 3], has previously been implemented in studying subjects such as pharmacology, rheumatology, surgery and radiology [4–9], and could be well placed to assist in studying neuroscience and neuroanatomy [10–12].

Electronic modules (eModules) are a form of CAL that describe digital learning packages that can integrate written subject content with multimedia graphics, interactive questions, tailored feedback and clinical cases [13]. In neurosciences education, previous studies have shown that these features increase user enjoyment, motivation and test performance compared to traditional forms of teaching [14–16].

Another form of CAL is the usage of online-accessible websites with medical information, either consisting of user-generated, traditional peer-reviewed content or a mixture of both, as an educational source [17]. One of the most commonly used is the user-generated content website: Wikipedia (Wikimedia Foundation, Inc., Florida, United States) which is used by up to 94% of medical students at certain institutions [18, 19]. Its main benefits are its accessibility and the range of topics available [17], but is distinguished from eModules by the web pages’ lack of interactivity, complex 3D graphics, virtual cases and reliable peer-review.

‘Neurophobia’, the fear of neuroscience, is common among both healthcare students and professionals [20, 21]. A large-scale survey in 2014 among UK medical students found neurology as significantly more difficult to learn as compared to other specialties [22]. Contributing to neurophobia is the perception that neuroscience and neuroanatomy is challenging to understand and a subject matter that is difficult to teach [10, 23, 24]. It remains uncertain how neuroscience can best be taught and learnt [25]. The strength of CAL, such as interactive graphical representations of complex anatomy and flexible access, has been suggested to be of benefit in teaching neurology [26].

Computer-assisted learning has been implemented across medical education, but it remains uncertain which specific methods improve learning outcomes in neuroscience and help in students overcoming neurophobia. No studies to date have compared different types of CAL resources in neuroscience. This study presents a novel, interactive eModule aimed at medical students that integrates virtual clinical cases to enhance learning of fundamental neuroscience concepts and carried out a randomized control study to determine its utility as compared to a Wikipedia-like source. The primary outcome in this study was the difference in perceptions related to engagement, usefulness and enjoyment between the two learning activities and the secondary outcome was the difference in learning efficacy.

### Methods

#### Participants

Participants were recruited from medical schools across London using administrative email lists and student social media groups. Recruitment was over the span of 7 months from March 2019 until September 2019. The inclusion criterion were any students enrolled at the time of recruitment in a U.K. medicine course and undergoing clinical placements. Excluded were healthcare professionals, medical graduates and students from disciplines other than medicine. Baseline characteristics were collected on the device used to access their allocated learning activity and previous exposure to clinical neurosciences. While all participants were medical students, some had greater exposure to neuroscience than others, since they may have completed a previous degree in neuroscience before their medical studies. For this reason, students who had previous experience at graduate level were categorised as "neuroscience postgraduate", at undergraduate level: "neuroscience undergraduate", those with no prior higher neurosciences education: "medical undergraduate”, and those that had not yet undertaken a neuroscience placement as a medical student as “secondary school”.

#### eModule design

**Fig. 1:**
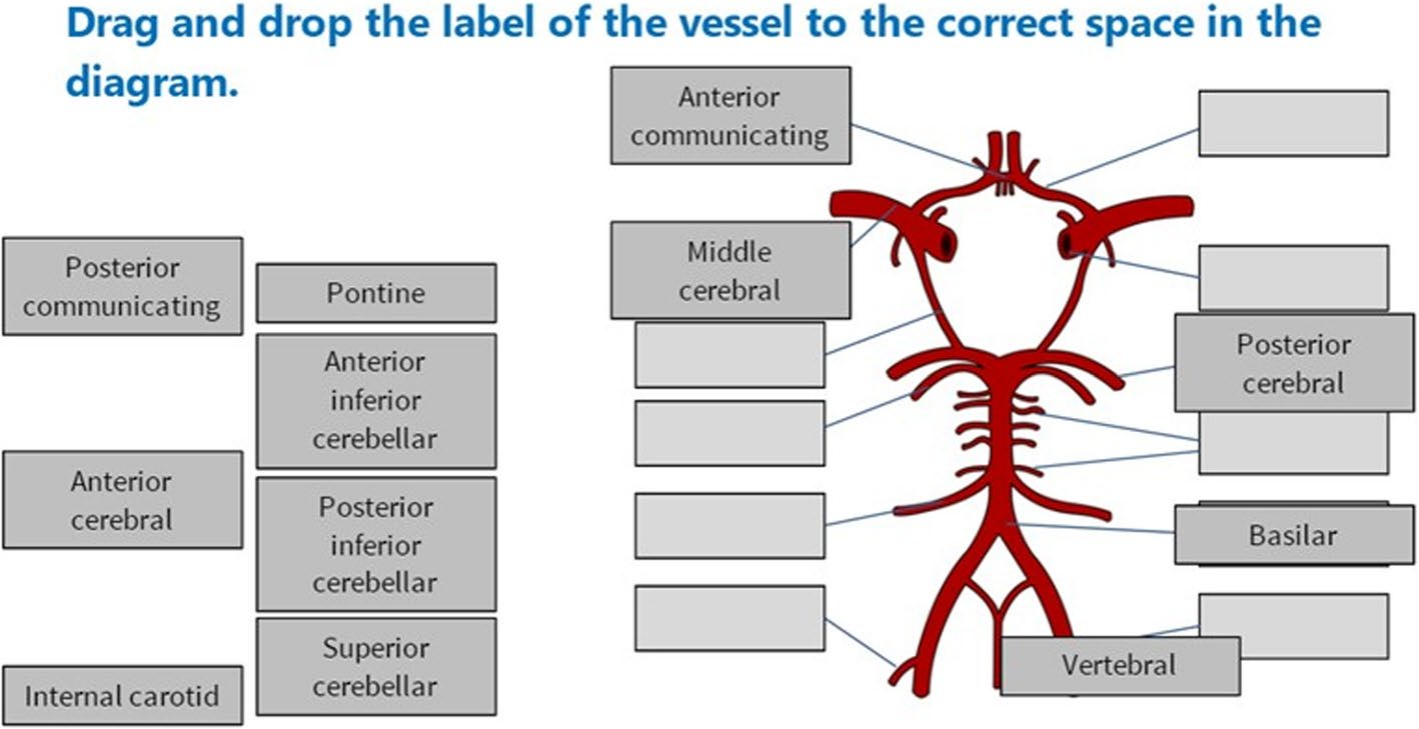
Screenshot of the eModule. An example of the drag-and-drop questions regarding the anatomy of the circle of Willis. The schematic representation of the Circle of Willis was made by Rhcastilhos [29] and released into the public domain

The eModule on stroke and the cerebrovascular anatomy was part of a series of neuroscience modules that were designed and developed by medical students over the span of 6 months with multidisciplinary input. This included a senior neurology professor, an educational specialist and was supervised by an academic neurosurgical fellow [27]. It took an estimated 60 h to fully develop the module. The learning objectives were to recognise common presentations of a stroke, know the anatomy of the cerebral arterial system, understand the relationship between the arterial system and clinical presentation and understand the core clinical management for stroke. A case-based approach with clinically relevant neuroimaging was used to teach cerebrovascular pathology, the cerebral arterial system and was designed to take approximately 20 to 30 min to complete. The computer software Storyline 360 (Articulate Global, Inc, New York, NY) enabled interactive features: drag-and-drop, multiple-choice and click-and-point questions, and the module was accessible on Android, iOS and all flash supporting web browsers. Storyline 360 is part of an Articulate 360 subscription and costs $649 per academic user per annum [28]. All images used in the module were obtained from Creative Commons license sources. Figure 1 shows a screenshot of the eModule and a weblink and additional screenshots are provided in the Supplemental Material.

#### Wiki design

**Fig. 2:**
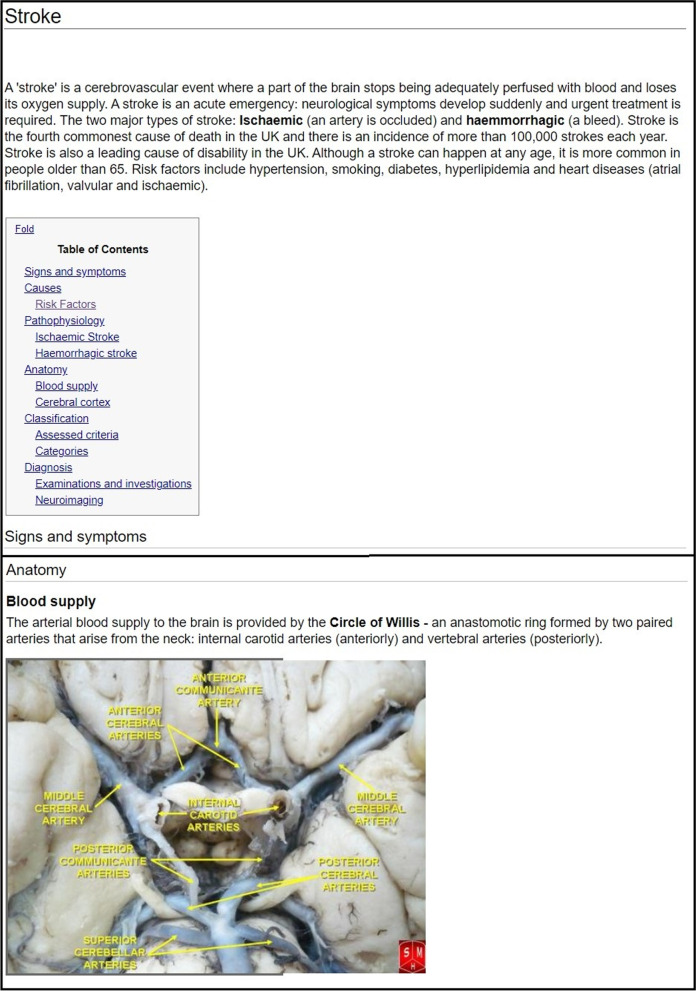
Screenshots of the Wiki page, highlighting the factual Wikipedia-like delivery and structure of the information on stroke and circle of Willis. The photo of circle of Willis is published with Creative Commons Attribution-Share Alike 3.0 Unported licence [30]

As part of the study, a Wikipedia-style page was created as a ‘control’ CAL activity using Wikidot.com (Wikidot Inc., Torun, Poland). The content of the Wiki page was identical to the eModule: including use of the same images, but without interactivity or clinical cases. The design of the Wiki page was based on the structure used in medical articles on Wikipedia. The creation of the Wiki page took an estimated 10 h. Figure 2 provides screenshots of the Wiki page and a weblink is provided in the Supplemental Material.

#### Study protocol

The aim of this study was to compare the efficacy and enjoyability of an interactive eModule to a ‘passive’ Wikipedia-like webpage. To test the previously described additional features of the eModule, participants were randomized using an allocation sequence to either the designed eModule or the Wiki group at the point of enrolment by only the first author as part of the study protocol. After randomization, participants were asked to complete their assigned intervention, immediately followed by a survey and quiz. After anonymized data extraction, the study authors were blinded to group allocation. Both groups were asked to complete the module on a device of their choice at a time convenient for them. It was estimated the learning portion for each group would take 20 to 30 min. Time spent on either learning activity were self-reported by participants as the different software used did not allow for objective time measurement. All participants were asked to complete a second quiz after 8 weeks to assess retainment of knowledge.

#### Study outcomes

The primary outcome measured in this study was the participants’ perception of their completed intervention via an online survey. For this, a five-point Likert scale, ranging from strongly disagree to strongly agree, was used to assess on how (1) enjoyable, (2) engaging, (3) recommendable and (4) useful the learning activity was perceived. Additionally, participants were asked to state which aspects of the intervention they found aided their learning, in order to assess if the matched content was found to be similarly useful between the two learning activities and to measure the proportion of the eModule group finding its unique features beneficial. The survey design was based on feedback forms used previously on the learning platform of the eModule’s design team, and reviewed by the educational specialist as part of the eModule’s implementation. To test if the two learning activities were accessed similarly, participants were asked what device was used to complete the intervention and the time spent on the intervention.

The secondary outcome was the efficacy of the learning activity, assessed using an electronic quiz completed immediately after the intervention. This quiz consisted of ten multiple-choice questions with a single best answer based on the content taught in the module, resembling the written exams commonly used in medical courses. The questions and answers of the quiz were designed by the same team who developed the eModule including a consultant neurologist and professor of neurology (both who previously, have been part of institutional faculty in setting formal examination questions). The aim was to assess the stated learning objectives of the learning activity. The same combination of questions were used in each quiz: several involving simple recall, some short case-based questions requiring 2-stage recall and a few harder questions requiring recall from multiple sources within the learning activities and weighing up of this information to determine an answer. A pre-specified answer grid of correct responses was created for each quiz. No negative marking was used, and the score is presented as the proportion of questions, answered correctly. A second quiz was sent after 8 weeks to assess retention and consisted of questions that assessed the same topics of the first quiz but with changes made to the details of the questions. The quiz was validated during a preliminary study of the eModule with 14 medical students and junior doctors, showing knowledge improvement post-completion of the activity compared to pre-completion. Participants of the preliminary study were excluded from the study reported in this paper.

#### Statistical analysis

The Fisher’s exact test was used to assess statistical differences in demographical information. The statistical difference of Likert-scale survey scores was assessed by using the Mann–Whitney U test, assuming ordinal data. Reliability of the survey was assessed by calculating the Cronbach’s Alpha, considering a coefficient of 0.7 or higher as acceptable internal consistency [31]. An unpaired two-tailed two-sample t-test was used to assess the statistical difference of exam results between the two groups at each time-point. Additionally, a paired two-tailed two-sample t-test was used to assess statistical difference of the score between the first and second quiz within each group. Statistical significance was defined as a p-value < 0.05. A sample size of 15 was calculated to be required to detect a large difference in knowledge retention with a power of 80%, assuming an effect size of 0.8 or higher, between the first and second quiz in each group [32]. To adjust for the effect of potential confounders: previous neuroscience experience and the device used to access the learning activity, an analysis of covariance (ANCOVA) was performed. This tested the effect of the learning activity on the results of the first quiz, second quiz and the difference between the two quizzes while accounting for these variables.

#### Ethics

Ethical approval for this study was registered by King’s College London as minimal risk (MRS-18/19–8122). Participation was voluntarily and none of the researchers were in a position of power or involved in the medical education of any participant. Informed consent was obtained through email from all participants. All data, including the quiz results, were only shared within the research team and not used for any purpose other than this study.

## Results

**Fig. 3:**
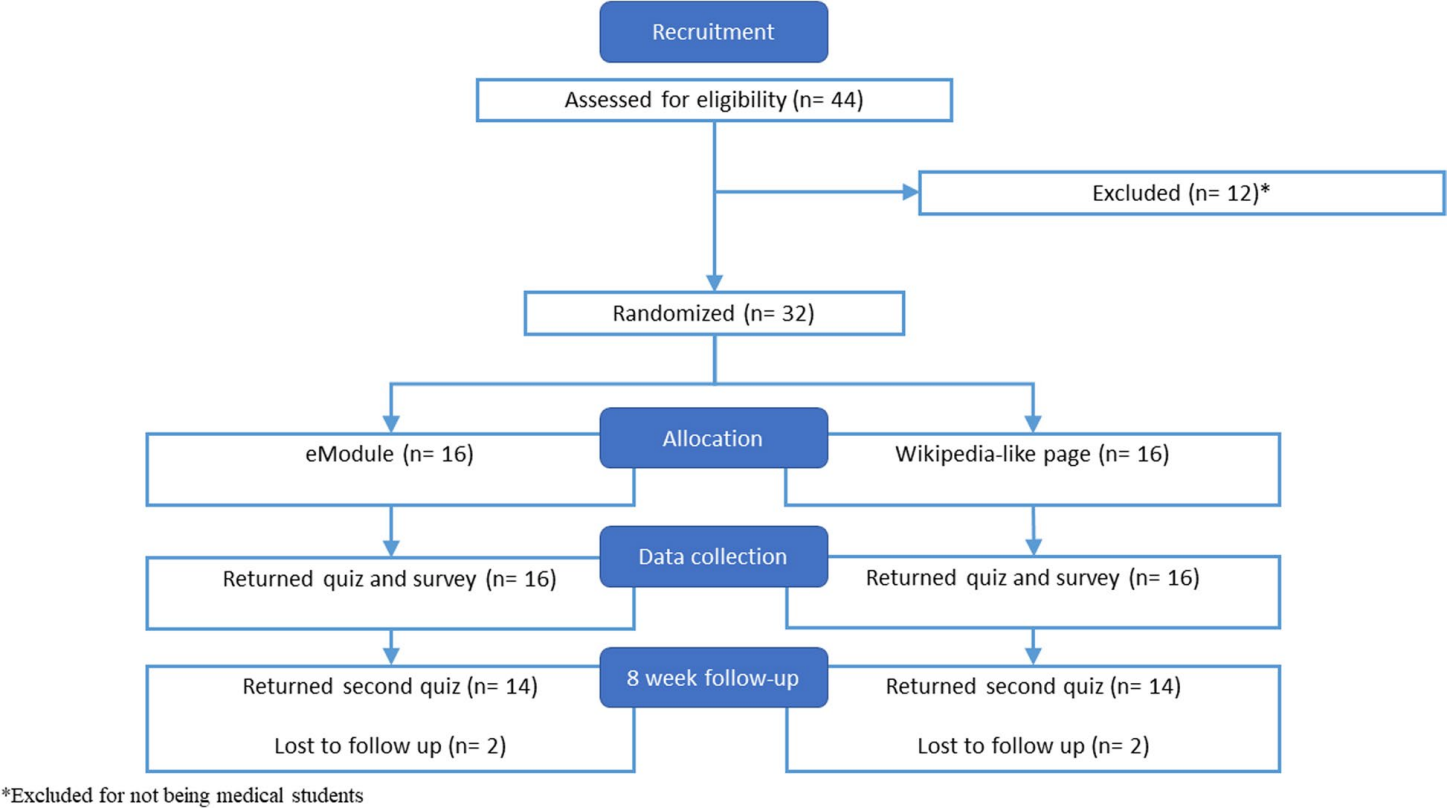
Participant flow diagram of the participants enrolled in the study

Thirty-two medical students participated in the study with 16 students in the eModule group and 16 students in the Wiki group. Twelve volunteers were excluded for not being a medical student. See Fig. 3 for the participant flow diagram. The electronic device used did not significantly differ between the two groups (*p* = 0.5) nor did their previous neuroscience exposure (*p* = 1), as detailed in Table 1. The eModule group spent a mean of 26 min (95% CI 16 to 36) on their activity compared to 17 min (95% CI 13 to 22) in the Wiki group, although this difference was non-significant (*p* = 0.1).

**Table 1:** Characteristics of the eModule and Wiki group

	**eModule group (%)**	**Wiki group (%)**	***p*-value**
Total	16 (100%)	16 (100%)	
Device used			
Laptop	13 (81%)	9 (56%)	*p* = 0.5
Tablet	0 (0%)	1 (6%)	
Phone	2 (13%)	3 (19%)	
Desktop	1 (6%)	3 (19%)	
Prior neuroscience exposure			
Secondary school	0 (0%)	1 (6%)	*p* = 1
Medical undergraduate	13 (81%)	12 (75%)	
Neuroscience undergraduate	3 (19%)	2 (13%)	
Neuroscience postgraduate	0 (0%)	1 (6%)	

In the eModule group, most participants found the clinical case studies and interactivity aided their learning and a smaller majority found the graphics, content and structure useful. In the Wiki group most participants found the graphics aided their learning while again a smaller majority found the content and structure useful. There was no significant difference between participants of the eModule and Wiki group in finding the graphics, content and structure aiding their learning (Table 2). The Cronbach’s alpha was 0.81 for the survey, indicating acceptable internal consistency.

**Table 2:** Aspects aiding learning according to participants

	**eModule group (%)**	**Wiki group (%)**	**p-value**
Graphics	12 (75%)	13 (81%)	0.7
Content	11 (69%)	9 (56%)	0.5
Structure	9 (56%)	9 (56%)	1
Clinical case studies^a^	13 (81%)	-	-
Interactivity^a^	14 (88%)	-	-

^a^Interactivity and clinical case studies were only used in the eModule and thus not asked about in the Wiki group

**Fig. 4:**
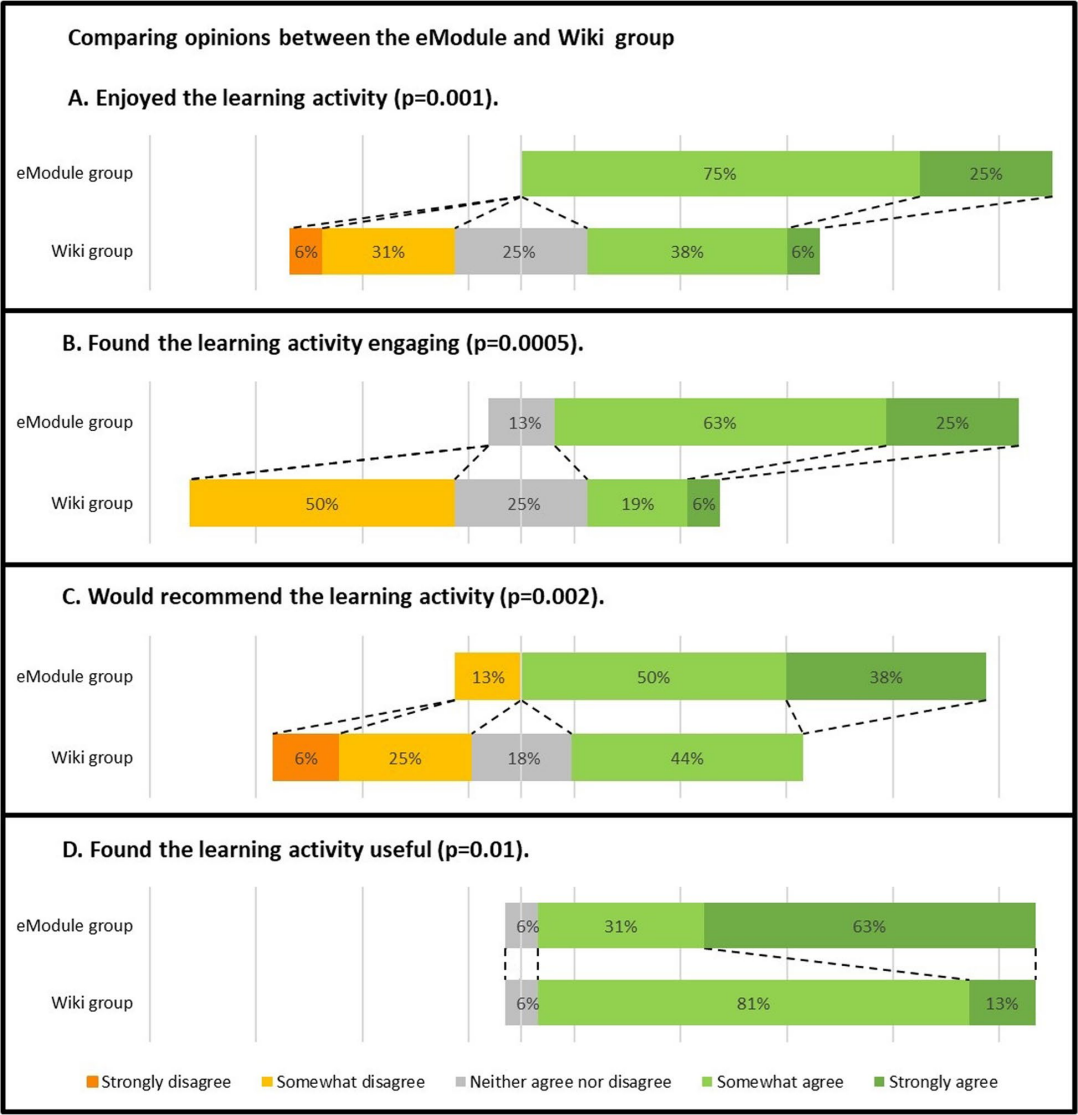
Comparing opinions between the eModule and Wiki group by comparing the eModule and Wiki group survey results of the Likert scale responses to the following statements. Proportions are given per option

The eModule group found their learning activity significantly more enjoyable (*p* = 0.001), engaging (*p* = 0.0005), recommendable (*p* = 0.002) and useful for their studies (*p* = 0.01) as compared to the Wiki group. These results are shown in Fig. 4.

The mean score of the first quiz was 86% (95% CI 82% to 91%) for the eModule group and 85% (95% CI 76% to 94%) for the Wiki group with no evidence of a statistical difference (*p* = 0.8). Fourteen participants in each group returned the second quiz after a mean of 59 days. The four participants not returning the second quiz did not significantly differ in previous neuroscience experience (*p* = 0.6).

**Fig. 5:**
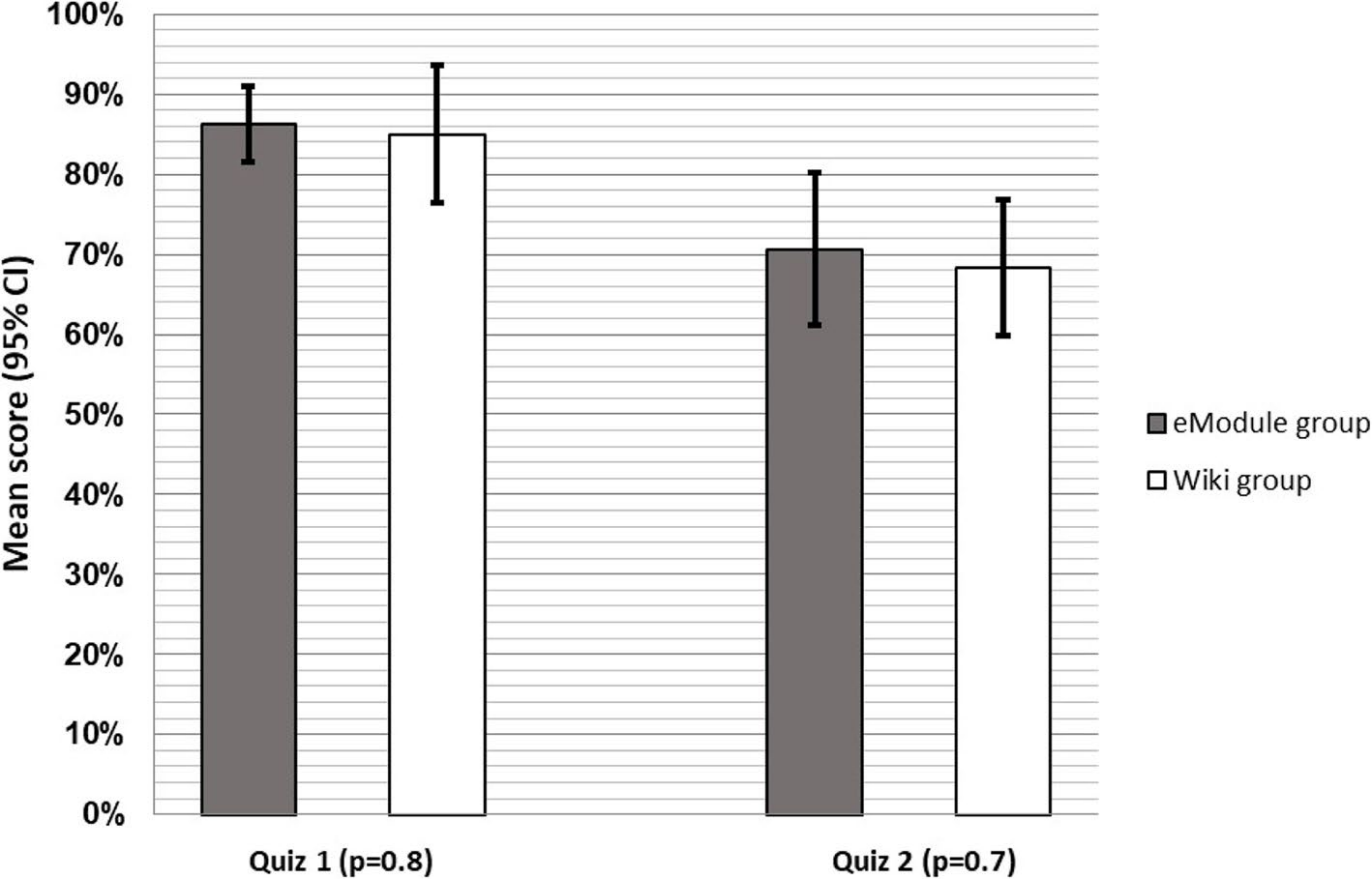
Quiz scores of eModule and Wiki Group. Mean proportional quiz scores (95% CI) and statistical difference between the eModule and Wiki Group for the first and second quiz. The first quiz was done immediately after the learning activity and the second quiz was sent 8 weeks later

There was a significant mean decline in the second quiz scores for both the eModule group (-16%, 95% CI -8% to -23%, *p* = 0.001) and Wiki group (-17%, 95% CI -8% to -26%, *p* = 0.003). Differences between the mean score of the second quiz for the e-learning group (71%, 95% CI 61% to 80%) and the control-group (68%, 95% CI 60% to 77%) were non-significant (*p* = 0.7). The mean quiz scores are shown in Fig. 5. After adjusting for device used and previous neuroscience experience there remained no significant effect of the learning activity on the test score of the first quiz (F(1, 24) = 0.42, *p* = 0.5), second quiz (F(1, 20) = 0.78, *p* = 0.4), or difference between the first and second quiz (F(1, 20) = 0.01, *p* = 0.9).

## Discussion

The aim of this study was to investigate if the perceptions and effectiveness of a neuroscience electronic learning module differed to a Wikipedia-style webpage. Students perceived the eModule as more engaging, useful and enjoyable, and the majority found the interactivity and clinical cases aided their learning. In contrast there was no difference found in immediate or long-term learning retention in using the eModule as compared to a Wiki-page with the same subject matter.

The learning experience using the eModule was more positively perceived compared to studying using the Wiki-style webpage. Motivation and pedagogic guidance are required for students to engage in optimal self-directed learning in medicine [33, 34]. eModules are well placed to facilitate these qualities. The structure of an eModule allows educators to focus the learning of the students and provide real-time feedback. Their interactivity is important in recruiting active participation, depth of information processing and cognitive engagement [35]. Enjoyableness in e-learning has previously been positively associated with deep learning [12], although this does not always translate to greater participant usage and uptake [36, 37]. Barriers to engagement with CAL are misaligned expectations, overwhelming volume of content and perceptions of being a passive recipient [38]*.* Greater motivation through the increased enjoyment and engagement of eModules may facilitate further learning and knowledge seeking behavior, but this overall effect is hard to assess.

In our study, the eModule group found their learning activity was more engaging, useful and enjoyable, but there were no significant group-wise differences with respect to perception of content, graphics or structure as adjuncts to assist learning. This suggests that the unique eModule features, namely, clinical case studies and interactivity (which represent the differentiating aspects between CAL methods) contributed the most toward a positive learning experience. Case-based teaching, which integrates theoretical neuroscience with ‘real’ clinical neurology, has previously been well documented to alleviate neurophobia [39]. Similarly, the interactivity of CAL by using multiple-choice questions and clickable graphics, is found to correlate with satisfaction and student engagement [40, 41], and that increased user engagement can be associated with increased knowledge scores [42]. Indeed, it is argued that interactive features and the aesthetic medium of a CAL platform can facilitate the understanding of difficult neuroscience and neuroanatomical concepts [10, 24]. In this study, it was likely that the eModule’s calibrated structure and use of interactivity sustained user attention and is therefore better suited for self-directed learning as compared to using passive resources. However, while these features were self-reported as being helpful, they did not translate into higher test scores.

That similar quiz scores were achieved in both groups could have been attributable to the quality of the subject content. The text and images; same in both learning activities, may have been of sufficient value to convey the learning aims without the interactivity and case studies being of additional benefit. One study, comparing three CAL modules on anatomy and physiology of the liver and biliary system with the same content but different levels of interactivity, found that students using the most passive and least interactive medium scored higher in their test compared to the other groups [43]. Similarly, the addition of complex psychosocial clinical cases to web-based modules on ambulatory medicine did not result in increased knowledge test scores for internal medicine residents, despite them finding them valuable [44]. In a meta-analysis by Cook et al. [42], across five studies comparing different modes of CAL it was found that increased user interactivity led to longer participant engagement time [44–48]. But this additional time was not associated with improved test scores. These studies demonstrate, at least in part, that controlling the interactivity and medium of the learning activity has less influence on learning efficacy than expected. It is possible that student engagement on CAL may not always involve active attention on the subject matter: loading software elements, passive watching of videos or clicking to progress through the modules are examples where participants might switch to ‘autopilot’ [49, 50]. Although in our study, participants stated the interactive elements aided their learning experience, some ill-implemented elements in the eModules could be distracting to some users and offset any gained learning potential.

Despite this, some individual randomized controlled trials have identified specific forms of interactivity that were, to a certain extent, associated with improved test outcomes. For example, the addition of case-based multiple-choice questions with feedback on an internet-based module for internal medicine residents was associated with significantly longer engagement time and higher test scores (78.9% ± 1.0 vs 76.2% ± 1.0, *p* = 0.006) as compared to the same modules without [45]. Similarly, a study of a neuropharmacology CAL module that used interactive assessment questions and pop-ups for pharmacology students resulted in higher exam scores compared to an online accessible text document with the same content [51]. Specifically, they showed that both the duration and distinct times accessing the CAL module were positively associated with higher test scores. Both studies suggest the benefit of well-designed interactive features is increased engagement resulting in higher test scores. Although there was a tendency of eModule users in this study to spend a longer time on it, this difference was not significant, nor the difference in the quiz scores.

In general, the results of this study are more similar to a larger meta-analysis by Cook et al. [52] which found a very small and inconsistent positive effect on knowledge outcomes by internet-based CAL compared with non-internet methods suggesting that content is more important than delivery. Further research should investigate, and describe more specifically, what type of interactivity utilized in CAL increases meaningful engagement and knowledge outcomes.

### Limitations

This study had a number of limitations. Firstly, group sizes were relatively small and there was a drop out of two participants in each group. Although the groups were large enough to assess statistical differences in the perceptions of the learning activity and knowledge retention within in each group, the low sample size could have meant that the study was underpowered to detect a small true difference in the quiz scores between groups. Additionally, participants not returning their second quiz had no significant difference in previous neuroscience experience or device used, suggesting there was no bias due to participants lost to follow up. Secondly, the question type of the quizzes was based on multiple-choice questions commonly used in medical written exams. These test knowledge retention but do not assess critical thinking, decision making or dimensional understanding of neuroscience anatomy in detail. Higher levels of learning could be tested with more comprehensive assessments. Lastly, only one time point, after eight weeks, was used to test long-term retention of knowledge in this study. The rate of knowledge attrition is perhaps different between the learning activities—in the future this could be assessed using additional timepoints.

### Implications

Modern medical students and doctors are required to be self-directed life-long learners [53]. In practice, many medical students and doctors use Wikipedia and other websites as complementary learning resources [17]. The user-generated nature of Wikipedia brings up the question of accuracy [17], but the main benefits of easy accessibility, user-friendliness and vast amount of content already attract the majority of students [18, 19]. With CAL, students can choose the content, time, place and pace of their learning [54]. Vice-versa, medical educators can use specific CAL packages to deliver standardized and accurate teaching to students and trainees across different hospital placements or even universities. This is particularly relevant in teaching clinical neuroscience, as one third of medical schools in the United Kingdom are unable to guarantee teaching from a certified neurologist [55]. Developing interactive learning modules comes with opportunity costs, including the development, delivery and maintenance [54]. As this study shows that since learning efficacy does not differ, medical educators should evaluate if the costs of developing and utilizing eModules are justified. Other alternatives include curating and vetting already existing web-based learning resources. Indeed, students and junior doctors report difficulties finding reliable websites among countless online medical resources [17, 56]. The results of this study would suggest that if easy access is combined with high quality and professional reviewed content, a text- and graphic-based Wikipedia-style website might be as effective as more sophisticated and expensive interactive CAL modules.

Computer-assisted learning is increasingly being implemented in clinical neurosciences education [26]. The principal design aim of this eModule was to tackle neurophobia by integrating basic neuroscience and neuroanatomy with relevant interactive clinical cases. The difficulty of understanding basic neurosciences and its integration with clinical neurology, has been reported by students and doctors to be major contributors to neurophobia [57]. Indeed, a study of Irish medical students found that less than one percent found they learned the most from online resources as compared to over seventy percent from bedside tutorials [58]. The addition of web-based multimedia to their neuroscience curriculum in other medical schools was found by students to be a useful addition to traditional lecture-based learning [59, 60]. Our study demonstrates that having interactive elements and a case-based approach can aid student learning. Examples of interactivity here included, but were not limited to, the drag-and drop image of the Circle of Willis and dynamic highlighting of salient findings on CT head scans i.e. where correlation between basic science, patient and clinical information is pivotal for understanding. In this way, Storyline 360 and other similar interactive learning software are ideally placed to assist in learning clinical neuroscience. Other topics in this domain which would benefit from these features include lesions of both the peripheral nervous system and spinal cord and the neurological sequelae and investigations which are correlated with these.

Perhaps the most successful approach in terms of both learning efficacy and satisfaction, would be to take a blended model that mixes CAL with traditional neuroscience learning methods [14, 61, 62]. As this study indicates, while different forms of CAL can be equally effective – their cost and preparation are significantly different. This can help inform medical educators choose which method of CAL to use in their curriculum according to the resources (both financial and human) that are available and whether to target efficacy or student enjoyability. Further research should be conducted to gain a more in-depth understanding of: (i) how current medical students view and access available forms of CAL; (ii) which specific elements of CAL they find helpful and (iii) how these can be improved. Through qualitative research, the future implementations of CAL can be developed to better fit the needs of students.

## Conclusion

This study shows that an interactive and virtual case-based computer-assisted learning in the form of an eModule on stroke and the cerebrovascular anatomy is perceived as more engaging and useful than a Wikipedia-style webpage with matching content. There was a significant decline in knowledge retention after both learning activities. However, their effectiveness in both short and long-term learning did not appear to differ. As the trend in medical-schools continues toward e-learning, these results are helpful in understanding where this software are best placed in their curricula. These results suggests that as a teaching supplement, a webpage with similar content can be as effective as more sophisticated modules. On the other hand, more enjoyable learning modules could motivate more students to be active self-directed learners. Educators should weigh up if these modules are cost-beneficial.

## Supplementary Information

**Additional file 1.** Weblinks to the eModule and Wikipedia-like page. Screenshots of the eModule.

## Data Availability

The dataset used and analysed during the current study are available from the corresponding author on reasonable request.

## Abbreviations

CAL: Computer-assisted learning
eModule(s): Electronic module(s)
