# Supplementary material for: Comparing computer-assisted learning activities for learning clinical neuroscience: a randomized control trial
Source: BMC Med Educ. 2022 Jul 3;22:522. doi: 10.1186/s12909-022-03578-2 (PMC9250740; doi:10.1186/s12909-022-03578-2)
Supplement: Supplementary file 1 — Additional file 1. Weblinks to the eModule and Wikipedia-like page. Screenshots of the eModule. [file 12909_2022_3578_MOESM1_ESM.docx]

# Supplementary Information

## Weblinks to the eModule and Wikipedia-like page

1. eModule: <https://360.articulate.com/review/content/e2ecf6a4-75ee-4a59-9065-9c06bcf9546f/review>
2. Wiki page: <http://neuroemodule.wikidot.com/wiki:stroke#toc2>

## Screenshots of the eModule

**Supplementary figure 1.** Screenshot of the eModule


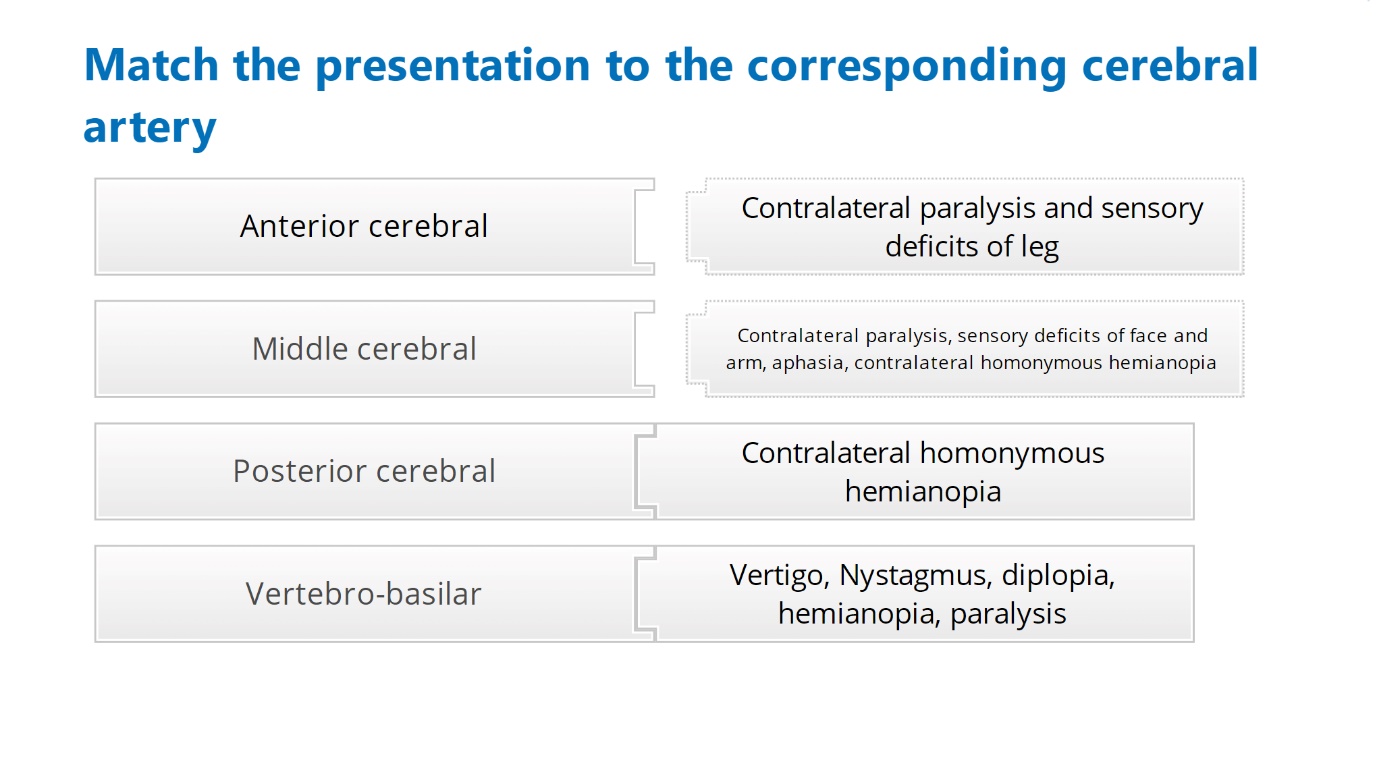


**Caption:** An example of a drag-and-drop question to match symptoms with the affected cerebral artery.

**Supplementary figure 2.** Screenshot of the eModule


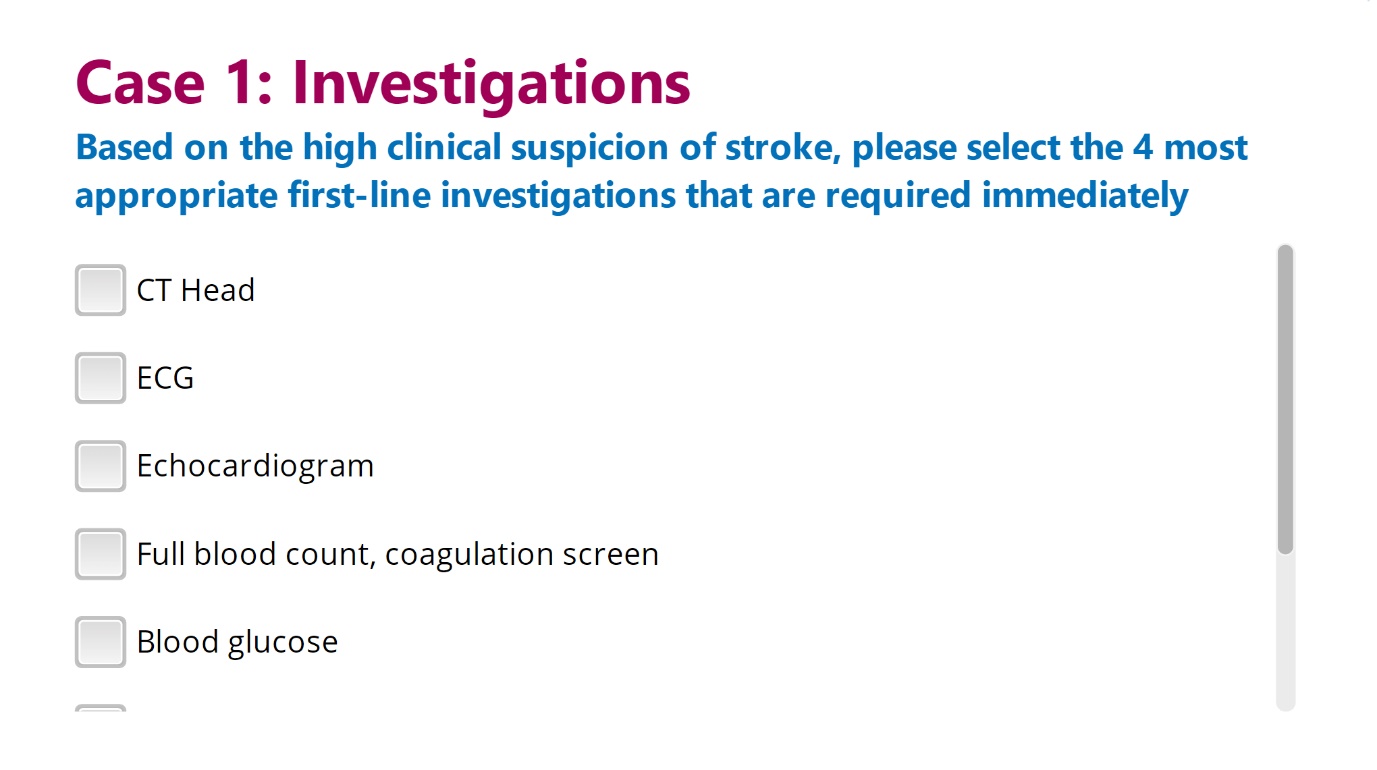


**Caption:** An example of a multiple-choice question asking about investigations required for the presented clinical case.

**Supplementary figure 3.** Screenshot of the eModule


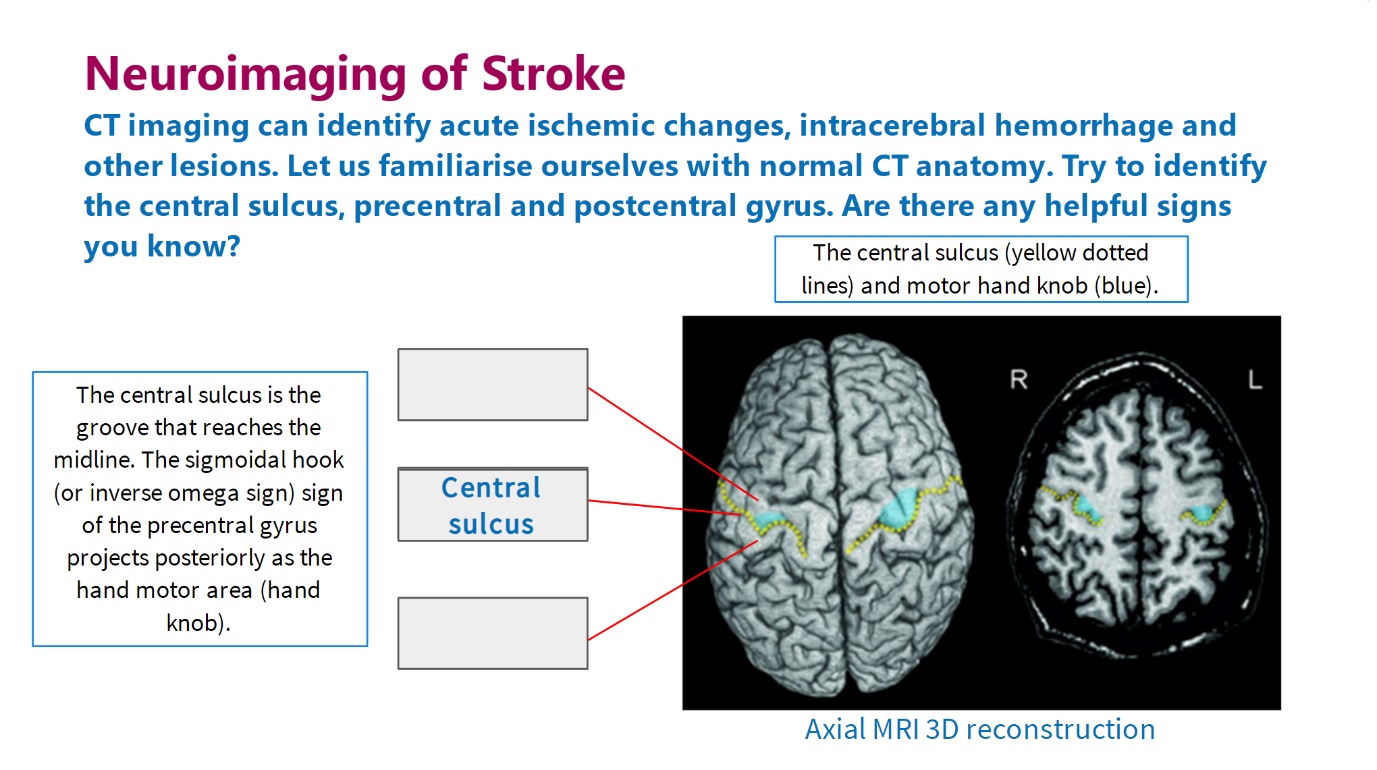


**Caption:** An example of an interactive image explaining the neuroanatomy of the central sulcus. The image showing the central sulcus and position of the motor handknob is published with Creative Commons Attribution 3.0 Unported licence (1).

# References in Supplementary Information

(1) Allen JS, Emmorey K, Bruss J, Damasio H. Position of the motor handknob and precentral gyrus. 2014; Available at: <https://commons.wikimedia.org/wiki/File:Position_of_the_motor_handknob_and_precentral_gyrus_fnana-07-00026-g001.png> Licence: <https://creativecommons.org/licenses/by/3.0/deed.en>. Accessed 26/02, 2022.
